# Nondestructive Evaluation of Soluble Solid Content of Cucumbers Based on VIS–NIR and SWIR Hyperspectral Images

**DOI:** 10.1002/fsn3.71055

**Published:** 2025-10-23

**Authors:** Fanghong Liu, Ning Zhang, Bo Huang, Xiujuan Chai

**Affiliations:** ^1^ State Key Laboratory of Robotics and System Harbin Institute of Technology Harbin China; ^2^ Agricultural Information Institute Chinese Academy of Agricultural Sciences Beijing China

**Keywords:** cucumber, hyperspectral imaging, SSC SWIR, VIS–NIR

## Abstract

Soluble solid content (SSC) is a key indicator for evaluating cucumber quality, directly influencing its commercial value. In China's cucumber sorting factories, SSC is typically assessed using random sampling and destructive methods, which are unsuitable for large‐scale and continuous detection. Therefore, this study employs hyperspectral imaging technology to evaluate the capability of visible–near infrared (VIS–NIR) and shortwave infrared (SWIR) spectroscopy for nondestructive SSC detection. In the experiment, hyperspectral data of cucumbers at different growth stages were collected in the VIS–NIR and SWIR. Using a partial least squares regression (PLSR) model, SSC prediction performance was compared across three spectral preprocessing methods and three sensitive wavelength selection methods. The optimal prediction models for SSC in the VIS–NIR and SWIR spectral ranges were established. The results showed that the optimal model in the VIS–NIR was the savitzky–golay smoothing (SG)‐fullwave‐PLSR model, with an R^2^_p_ of 0.827, an RMSEP of 0.176, and an RPD of 2.403. In the SWIR, the optimal model was the multiplicative scatter correction (MSC)‐competitive adaptive reweighted sampling (CARS)‐PLSR, with an *R* ^2^_p_ of 0.818, an RMSEP of 0.177, and an RPD of 2.344. The study demonstrates that hyperspectral imaging in both VIS–NIR and SWIR can be applied for nondestructive SSC detection in cucumber sorting factories. Considering both prediction accuracy and cost, VIS–NIR is more suitable for online monitoring of cucumber SSC.

## Graphical abstract

This study employs hyperspectral imaging (VIS–NIR/SWIR) for nondestructive soluble solid content (SSC) detection in cucumbers. Optimized PLSR models with preprocessing and sensitive wavelength selection achieved *R* ^2^_p_ of 0.827 (VIS–NIR) and 0.818 (SWIR). Results demonstrate that VIS–NIR and SWIR are viable for cucumber SSC detection, with VIS–NIR being more cost‐effective for online monitoring in sorting facilities.

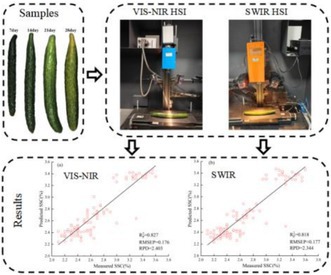

## 1 Introduction

China is the world's largest cucumber producer. Each year, it produces over 60 million tons of fresh‐eating cucumbers, accounting for 80% of the country's total cucumber yield (Gebretsadik et al. 2021). These cucumbers are known for their crisp texture, juiciness, and convenience. They align well with Chinese dietary preferences and have become a staple vegetable in the national diet (Zhang et al. 2021). Chinese consumers place great importance on the taste of fresh cucumbers, and SSC is a key indicator of flavor. Cucumbers with higher SSC taste better, last longer, and are more competitive in the market (Valverde‐Miranda et al. 2021). However, due to variations in growing conditions and individual differences, SSC in cucumbers can vary. Therefore, it is essential to implement non‐destructive SSC detection for fresh‐eating cucumbers. This ensures that cucumbers with high SSC are selected for the market. This approach meets consumer demand for high‐quality cucumbers and enhances their commercial value.

Hyperspectral imaging (HSI) technology can simultaneously capture both image and spectral information from the target sample. It offers advantages such as high detection speed, high accuracy, and nondestructive measurement (Wang et al. 2021). Researchers have primarily focused on the VIS–NIR and SWIR spectral ranges to study the quality of various agricultural products (Wieme et al. 2022). In the VIS–NIR, Sheng Gao et al. used spectral data (400–1000 nm) from red globe grapes to predict SSC. The optimal model, PCA‐PLSR, achieved a correlation coefficient of 0.976 (Gao and Xu 2022). Yun Chen et al. collected spectral data (400–1000 nm) from two wolfberry varieties using a hyperspectral imaging system. By applying various preprocessing and feature extraction methods, they developed an SSC prediction model with an *R* ^2^_p_ of 0.949 (Chen et al. 2024). Pan Tian et al. applied the SNV‐CARS‐PLSR model to spectral data in the 400–1000 nm for SSC prediction in mangoes, obtaining a prediction correlation coefficient of 0.9 (Tian et al. 2023). In the SWIR range, Jiangbo Li et al. developed an MC‐UVE‐SPA‐PLS model to predict SSC in pears. The model achieved a prediction root mean square error of only 0.35% (Jiangbo Li et al. 2016). Yujie Li demonstrated that SWIR hyperspectral imaging effectively predicts the SSC of jujube, with the prediction model *R* ^2^_p_ reaching 0.857 (Y. Li et al. 2022). These studies highlight the significant application potential of hyperspectral imaging in both spectral ranges for nondestructive SSC detection. They are also valuable methodologies for cucumber SSC evaluation research.

In studies on cucumber quality using hyperspectral imaging technology, Diwan *P. Ariana* et al. collected pickling cucumber spectral data in the 675–1000 nm range and combined it with a linear discriminant analysis (LDA) method to detect internal defects, achieving an accuracy of 99% (Ariana et al. 2006). Haiyan Cen et al. utilized two types of hyperspectral imaging modes within the 400–1000 nm range, integrating multiple machine learning methods to classify internal defects and chilling injury in pickling cucumbers. Their study achieved an accuracy of 95.1% for defect classification and 100% for chilling injury classification (Cen et al. 2014; Cen et al. 2016). Renlu Fu et al. collected spectral data in the 700–1000 nm range using a transmission mode and applied a PLSR‐DA model to detect fly infestation in pickling cucumbers, achieving an accuracy of 93% (Lu and Ariana 2013). These studies primarily focused on the VIS–NIR and qualitative analysis of the internal quality of pickling cucumbers, aligning with dietary habits in the local community and the demands of the local food processing industry. There are relatively few quantitative studies on the internal quality of cucumbers using spectral imaging technology. I. Kavdir et al. predicted cucumber dry matter content and firmness parameters (Area, Slope, Maximum force) using 550–1100 and 800–1650 nm spectral systems with PLSR modeling. The *R* ^2^_p_ was 0.65 for dry matter content, and 0.70, 0.67, and 0.52 for Area, Slope, and Maximum force, respectively (Kavdir et al. 2007). Kusumiyati et al. used a portable spectrometer (381–1017 nm) combined with a PLSR model to predict SSC and moisture content in six agricultural products from the Cucurbitaceous family, with the best prediction models achieving RPD values of 5.68 and 3.69, respectively (Kusumiyati et al. 2021). However, this model was developed for the Cucurbitaceae family as a whole rather than specifically for cucumbers. Moreover, the number of cucumber samples used was limited to only 50, and they were sourced from a single origin. Although interest in spectral‐based nondestructive techniques for cucumber quality assessment is increasing, most studies still focus on the qualitative analysis of internal quality in pickling cucumbers. The research on rapid and non‐destructive detection of SSC in fresh‐eating cucumbers based on hyperspectral imaging technology with different spectral wavelengths remains extremely scarce in current academic studies.

Currently, cucumber sorting factories in China mainly use destructive testing. A refractometer is applied to measure the SSC of randomly selected cucumbers. This method does not allow for large‐scale, continuous monitoring. To address this issue, this study systematically evaluates the performance of VIS–NIR (400–1000 nm) and SWIR (1000–2500 nm) hyperspectral imaging technologies for the non‐destructive detection of SSC in cucumbers. The goal is to provide scientific guidance for developing online detection methods and selecting suitable sorting sensors. For the first time, we constructed a dual‐band hyperspectral image dataset of cucumber samples at multiple growth stages, along with corresponding SSC measurements. The applicability of various preprocessing methods, including SG, MSC, and SNV, was evaluated for different spectral ranges, resulting in tailored preprocessing strategies. Further, characteristic wavelengths most sensitive to SSC were extracted using algorithms such as SPA, UVE, and CARS. Based on this, PLSR models for predicting cucumber SSC were developed and optimized for different spectral ranges. This study provides a solid data foundation and a refined modeling approach for non‐destructive SSC detection in cucumbers. To the best of our knowledge, this is the first study to systematically explore SSC prediction in cucumbers from a dual‐band hyperspectral perspective. It provides both theoretical support and technical reference for developing online detection methods and selecting parameters for sorting equipment.

## 2 Material and Method

### 2.1 Sample Preparation

The fresh‐eating cucumber (Jinyan No. 5) samples used in the experiment were sourced from the Hongji Agricultural Company in Zibo, Shandong Province. Cucumbers at the same growth stage tend to have SSC values concentrated within a range. To enhance diversity, we collected cucumbers at different growth stages as samples. We collected cucumbers at growth dates of 7, 14, 21, and 28 days, with 100 cucumbers from each stage, totaling 400 cucumbers. During the collection process, only cucumbers with good appearance, free from rot or defects, were selected.

### 2.2 VIS–NIR and SWIR Hyperspectral Image Acquisition

The VIS–NIR and SWIR hyperspectral imaging systems used in the study are models Y10E‐Zyla and N25E‐SWIR (ISUZU OPTICS Co. Ltd., China), respectively. Both hyperspectral imaging systems are placed in the same darkroom and include a hyperspectral spectrometer, computer, halogen lamps, and cameras. The cucumber samples were placed on a black background panel with a distance of 150 mm between the samples and the camera lens. The VIS–NIR system captures spectra in the range of 368–970 nm, covering the visible and near‐infrared regions of the spectrum. Due to high noise, the range of 368–400 nm was removed, and only the range of 400–970 nm was retained. The spectral resolution is 2.35 nm, with 240 bands in total, and the imaging resolution is 1024*1660. The SWIR system captures spectra in the range of 901–2517 nm, covering the entire short‐wave infrared region. For subsequent analysis, the 1000–2400 nm wavelength range was selected. The spectral resolution is 6.25 nm, with 288 bands in total, and the imaging resolution is 384*1840.

To eliminate the effects of light intensity variations and dark currents in the lens on imaging, and to calculate the relative reflectance spectra of the scanned object, a black‐and‐white board calibration was performed before each batch of cucumber spectral data collection (Baranowski et al. 2012). The equation is as below:

$$R=\frac{I_{R}-I_{D}}{I_{W}-I_{D}}$$

**FIGURE 1:**
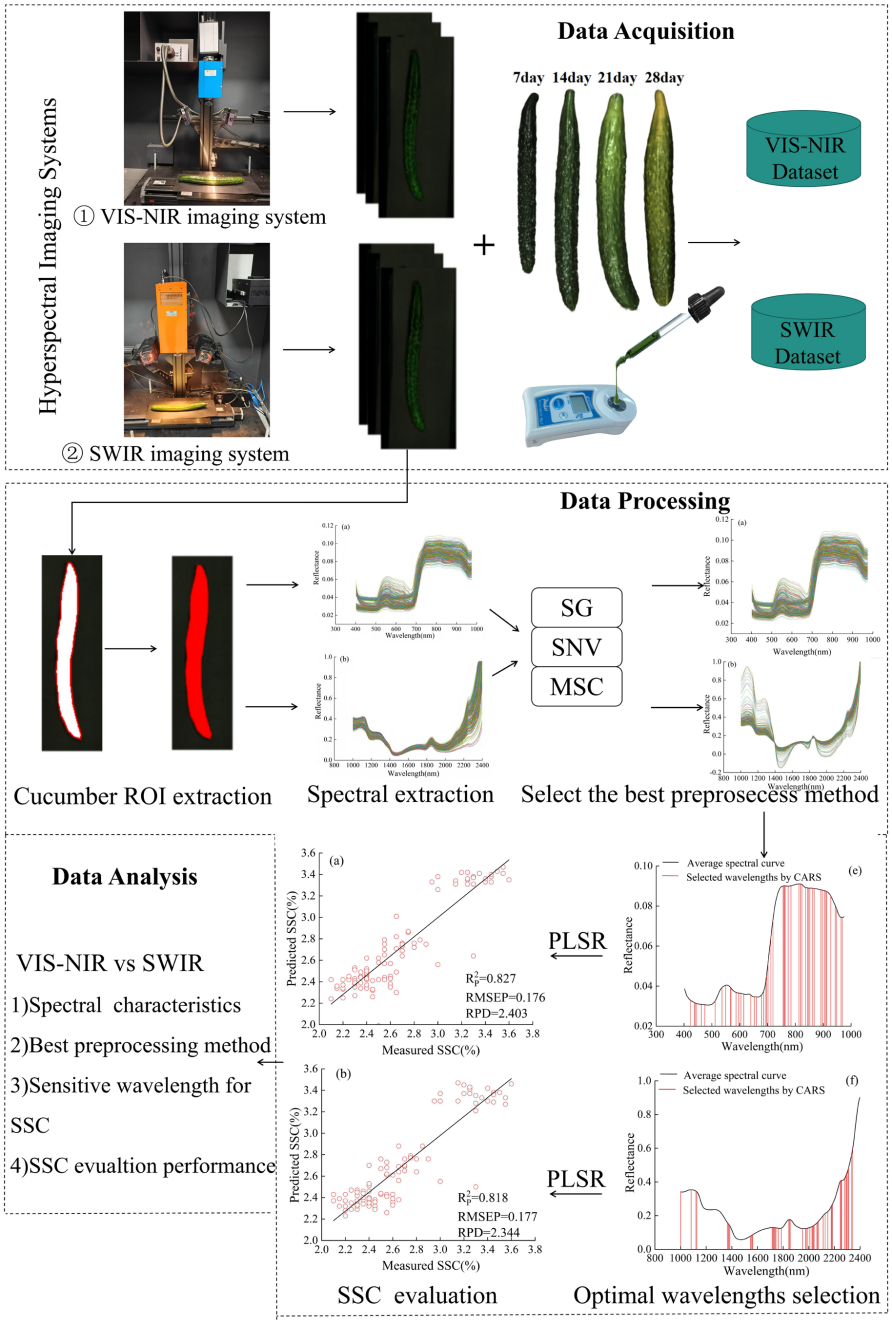
Overall technical workflow.

In the equation: R represents the calibrated image; I_R_ represents the raw image; I_D_ represents the blackboard calibration image; and I_W_ represents the whiteboard calibration image. For the calibrated spectral images, the entire cucumber was selected as a region of interest (ROI), and the average spectral reflectance of all pixels within each ROI was calculated. The overall technical workflow is illustrated in Figure 1.

### 2.3 Measurement of SSC

According to the NY/T 2637–2014 standard, the cucumbers were cut into small pieces and juiced using a juicer. The juice was then filtered using a filter to remove all solid particles, ensuring that they do not affect the refractometer reading. Before measurement, the digital refractometer (PAL‐1, Atago Co. Ltd., Tokyo, Japan) was calibrated with distilled water to ensure accurate readings. The filtered cucumber juice was then dropped onto the measurement window of the refractometer. The SSC displayed on the refractometer was recorded, usually expressed as a percentage (%). To ensure measurement accuracy, the refractometer window and all tools in contact with cucumber juice were cleaned immediately after each measurement.

### 2.4 Spectral Data Preprocessing

During the hyperspectral data acquisition, external environmental factors and instrument interference can introduce redundant information and noise, which reduce the model's stability and prediction accuracy (Li et al. 2020). To effectively reduce the impact of these disturbances on model performance and evaluate the applicability of different preprocessing methods within the VIS–NIR and SWIR, this study employed three spectral preprocessing techniques: Multivariate Scatter Correction (MSC), Standard Normal Variate transformation (SNV), and the smoothed Savitzky–Golay (SG) filter.

MSC corrects the scattering differences between samples, eliminating spectral baseline shifts and intensity variations caused by light scattering effects (Helland et al. 1995). SNV standardizes each spectral sample to remove baseline drift and optical path differences between samples, making the spectral data more consistent with a normal distribution (Barnes et al. 1989). The smoothed SG filter uses polynomial fitting within a local window to smooth the data and reduce noise while preserving spectral features such as peak values and bandwidths (Zhou et al. 2020).

### 2.5 Sensitive Wavelength Selection

Hyperspectral images contain high‐dimensional data that suffer from collinearity and redundancy between adjacent wavelengths, which not only reduces computational efficiency but also impacts model performance (Yuan et al. 2021). Therefore, selecting the most effective sensitive wavelengths, without losing key information from the original spectral data, can enhance both the accuracy and efficiency of the data. In this study, the Successive Projections Algorithm (SPA), Competitive Adaptive Reweighted Sampling (CARS), and Uninformative Variable Elimination (UVE) methods were employed to extract effective spectral features from the VIS–NIR and SWIR spectral data.

SPA identifies and eliminates redundant variables, addressing information redundancy and multicollinearity. This algorithm generates a candidate subset of variables with minimal collinearity through projection operations and ultimately selects the subset with the smallest Root Mean Square Error (RMSE) as the effective wavelengths (Huang et al. 2013). CARS combines stepwise regression and adaptive reweighting methods with Monte Carlo simulations to identify the optimal variable combinations. Through an iterative reweighting process, this algorithm dynamically selects the most influential variables for the model and ultimately chooses the subset with the smallest prediction error as the effective wavelengths (Jiang‐bo Li et al. 2014). UVE introduces random noise variables to assess the importance difference between the original and noise variables, thereby selecting variables with significant informational contribution. This algorithm calculates the stability index of each variable, removing those with little or no contribution to the model and ultimately retaining the subset of variables that significantly enhance the model's predictive ability (Lin et al. 2023).

### 2.6 Modeling Establishment and Evaluation

To compare the predictive performance of VIS–NIR and SWIR for cucumber SSC, PLSR models were developed. PLSR is a classical multiple regression algorithm that achieves linear curve fitting by minimizing the sum of squared errors and has been widely used in fruit and vegetable quality detection (Sun et al. 2017; Xiong et al. 2015; Zhou et al. 2018).

The evaluation metrics for regression models under different spectral include the coefficient of determination for the calibration set (*R* ^2^_c_), the coefficient of determination for the prediction set (*R* ^2^_p_), root mean square error of calibration (RMSEC), root mean square error of prediction (RMSEP), and residual predictive deviation (RPD). Values of *R* ^2^_c_ and *R* ^2^_p_ closer to 1 indicate higher model stability and goodness of fit. Smaller RMSEC and RMSEP values reflect stronger predictive capability. The RPD represents the relative predictive performance of the model. An RPD value between 2 and 2.5 suggests effective predictive performance, while a value between 1.5 and 2 indicates relatively poor predictive ability.

## 3 Result and Analysis

### 3.1 Statistics for SSC and Datasets Division

Table 1 shows the SSC of cucumbers at different growth stages (7 days, 14 days, 21 days, and 28 days). At 7 days, the average SSC content is the highest, with the largest variance, indicating that cucumbers at this stage have a better taste but exhibit significant individual differences. At 14 days, the SSC content decreases, and after 21 days, it tends to stabilize and remain constant. This phenomenon occurs because cucumbers are still in their rapid growth phase at 7 days, during which they accumulate sugars, resulting in the highest SSC. After 7 days, as the growth period extends, SSC content decreases, which may be attributed to factors such as increased water content and sugar metabolism (Davies and Kempton 1976).

**TABLE 1:** Table The SSC of cucumbers at different growth dates.

Growth dates (days)	Mean (%)	SD (%)	Maximum (%)	Minimum (%)	Representative image
7	3.34	0.21	4	2.85	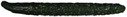
14	2.66	0.12	3	2.35	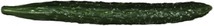
21	2.36	0.13	2.7	1.95	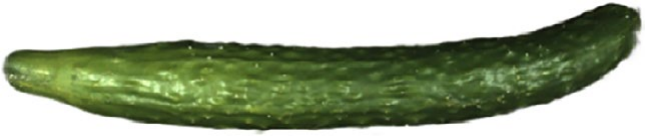
28	2.36	0.15	2.95	2.10	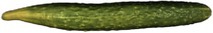

We used a stratified sampling algorithm to divide the raw spectra of 400 cucumber samples into a calibration set and a prediction set at a ratio of 3:1. The division results are shown in Table 2.

**TABLE 2:** Statistical analysis of SSC in calibration set and prediction set.

Index	Data set	Samples	Mean (%)	SD (%)	Max (%)	Min (%)
SSC	Calibration set	300	2.69	0.44	4.00	1.95
	Prediction set	100	2.67	0.41	3.60	2.10

As observed in the table, the mean and standard deviation of the calibration and prediction sets are similar, and the distribution range of the prediction set falls within that of the calibration set. This indicates that the stratified sampling algorithm is appropriate.

### 3.2 Comparison of Spectral Characteristics on VIS–NIR and SWIR

**FIGURE 2:**
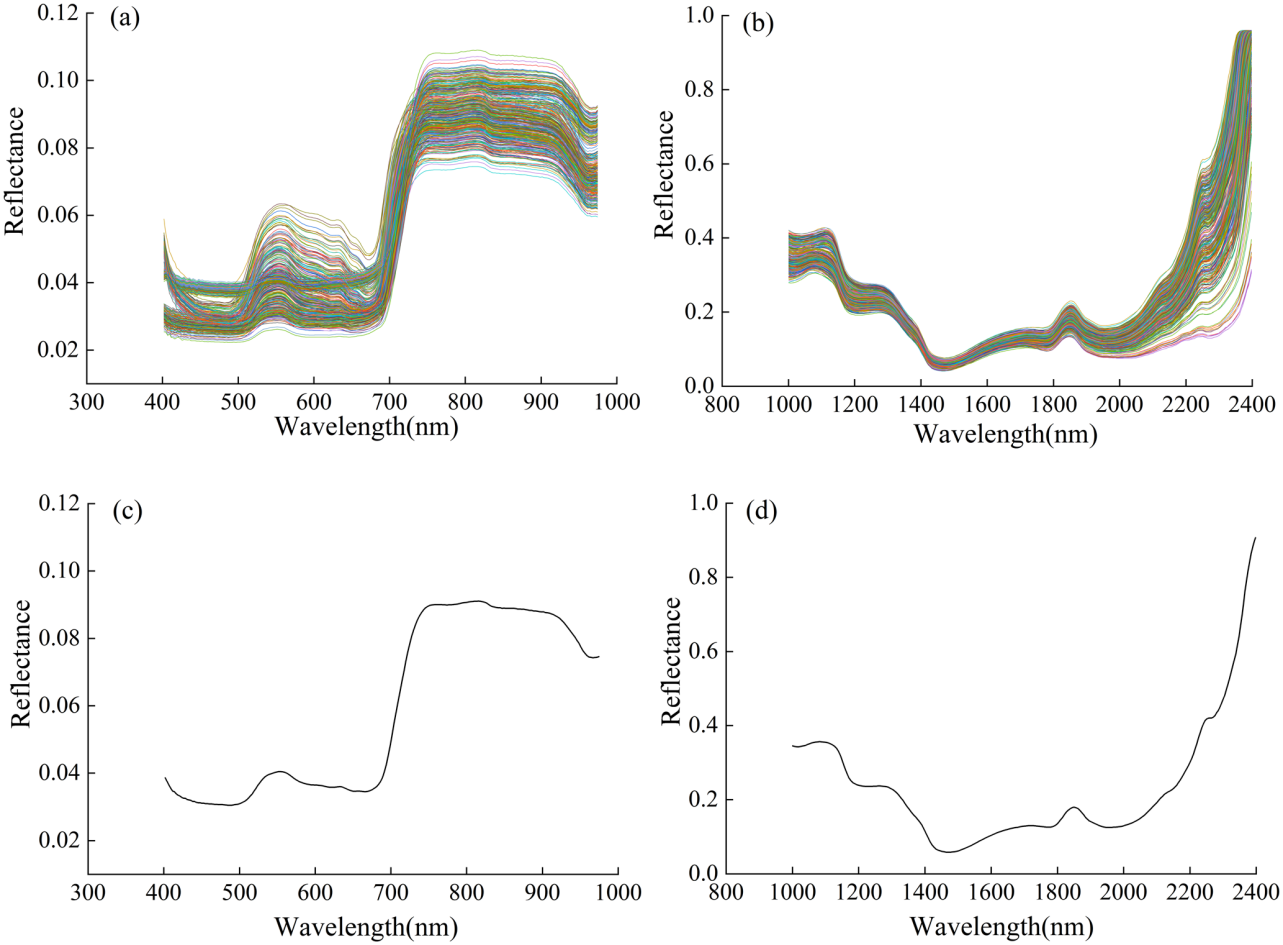
(a) Reflectance curve of cucumber on VIS–NIR; (b) Reflectance curve of cucumber on SWIR; (c) Average spectral curve on VIS–NIR; (d) Average spectral curve on SWIR.

In Figure 2a,c represent the reflectance image and the average reflectance curve of cucumber samples in the VIS–NIR. As shown in (c), the trough observed at 400–500 nm reflects the absorption characteristics of carotenoids and lutein (Nicolaï et al. 2007). The peak at 550 nm corresponds to the green light region, where cucumbers exhibit enhanced reflectance due to their green appearance. The absorption peak at 680 nm is a characteristic chlorophyll absorption feature (Cho et al. 2021). The reflectance increases in the 700–750 nm range, marking the red‐edge effect. The trough around 960 nm is attributed to the combined effect of the second overtone of the O‐H bond in carbohydrates and water (Guo et al. 2016). In Figure 2b,d depict the reflectance image and the average reflectance curve of cucumber samples in the SWIR. As shown in (d), the reflectance peak in the 1000–1150 nm range corresponds to the C‐H and O‐H functional groups in major sample components such as water, sucrose, and cellulose (Sun et al. 2017). The troughs at 1200, 1450, and 1950 nm indicate water absorption regions. In the 2000–2400 nm range, water absorption has a relatively smaller effect, leading to a gradual increase in reflectance. The 2000–2400 nm range is characterized by combination vibrations of C—H, N—H, and O—H bonds (Prananto et al. 2020).

A comparison of (c) and (d) in Figure 2 reveals that the overall reflectance values in the SWIR are higher than those in the VIS–NIR. This phenomenon is primarily related to the optical, chemical, and physical structural properties of cucumbers. In terms of optical and chemical composition, the 400–700 nm is the main absorption region for pigments, whereas in the near‐infrared range, the absorption effect of pigments is almost negligible. Structurally, the tissue composition of cucumbers, including cell walls and intercellular spaces, causes stronger light scattering in the near‐infrared region, leading to increased reflectance. This scattering effect is particularly evident in the 1000–2400 nm because photons in this region have lower energy and greater penetration depth, making them more susceptible to scattering. Additionally, since the samples are spiny cucumbers with an uneven surface and a waxy layer, their external structure further enhances light reflection in the near‐infrared range, contributing to higher reflectance values.

### 3.3 Different Spectral Preprocessing Methods on VIS–NIR and SWIR

Three spectral preprocessing methods, MSC, SNV, and SG, were applied to preprocess the VIS–NIR and SWIR spectra of cucumber samples. Based on the PLSR method, calibration models were established for both spectral ranges, and the model performance was validated using an independent prediction set to determine the optimal preprocessing method for the two spectra. Table 3 presents the modeling results of the three preprocessing methods for the PLSR model under different spectra, including key evaluation metrics such as *R* ^2^_c_, RMSEC, *R* ^2^_p_, RMSEP, and RPD.

**TABLE 3:** Correction and prediction effect of PLSR model under different preprocessing methods on VIS–NIR and SWIR.

Wavelength	Pretreatment	Latent variables	Calibration set		Prediction set		RPD
			*R* ^2^_c_	RMSEC	*R* ^2^_p_	RMSEP	
VIS–NIR (400‐970 nm)	Raw	18	0.889	0.146	0.817	0.179	2.340
	MSC	16	0.886	0.148	0.804	0.184	2.258
	SNV	10	0.867	0.160	0.812	0.179	2.308
	SG	16	0.868	0.159	0.827	0.176	2.403
SWIR (1000‐2400 nm)	Raw	20	0.892	0.143	0.810	0.181	2.298
	MSC	15	0.869	0.158	0.817	0.178	2.340
	SNV	15	0.869	0.158	0.813	0.179	2.318
	SG	12	0.861	0.163	0.802	0.185	2.249

As shown in Table 3, the original spectral data in both spectral ranges exhibit good modeling performance, and the model performance changed to varying degrees after applying different preprocessing methods. In the VIS–NIR, the model after SG performed the best on the prediction set, with an R^2^p of 0.827, RMSEP of 0.176, and RPD of 2.403. SG significantly improved the model's prediction performance, indicating that this method effectively removed noise from the spectrum while retaining valuable spectral information. In the SWIR, the model after MSC treatment showed a significant improvement in prediction performance, with an *R* ^2^p of 0.817 and RPD of 2.340, both reaching the highest values. This indicates that MSC can effectively reduce the interference of scattering effects on the spectrum, improving the prediction accuracy of the model.

In summary, SG and MSC demonstrated the optimal preprocessing effects in the VIS–NIR and SWIR, respectively, enhancing the predictive ability of the models. Subsequent band extraction and modeling in VIS–NIR and SWIR will be performed using the corresponding optimal preprocessing methods.

### 3.4 Different Spectral Sensitive Wavelength Selected Methods and Model Establishment

**FIGURE 3:**
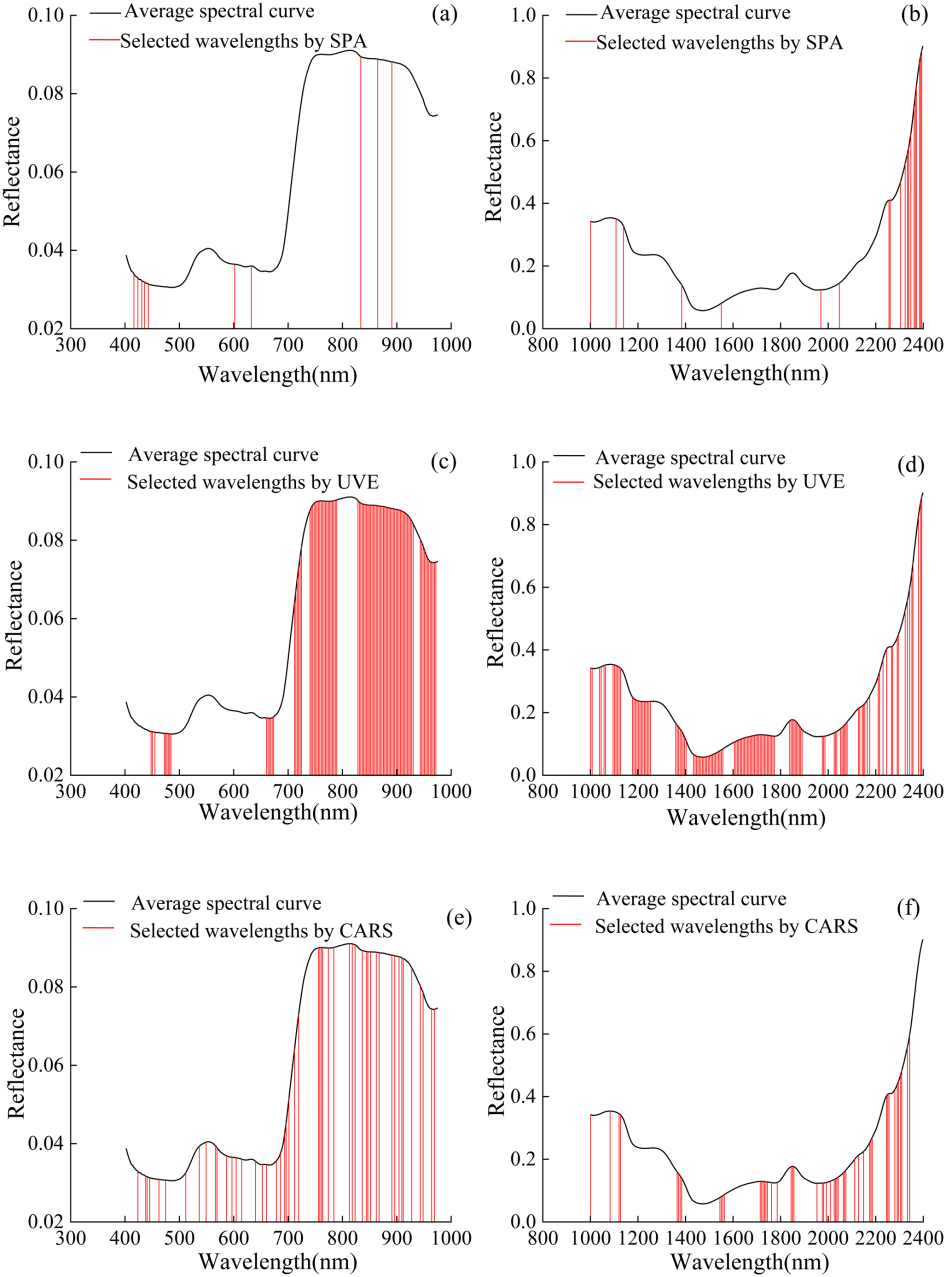
Selected sensitive wavelengths (a) SPA on VIS–NIR (b) SPA on SWIR (c) UVE on VIS–NIR (d) UVE on SWIR (e) CARS on VIS–NIR (f) CARS on SWIR.

The preprocessed VIS–NIR and SWIR underwent sensitive wavelength selection using various methods, including UVE, CARS, and SPA. The results of the sensitive wavelength selection for VIS–NIR and SWIR are shown in Figure 3. The red vertical solid lines below the average spectral curves represent the sensitive wavelengths extracted from the cucumber spectra that are related to SSC. Figure 3a,c,e, represents the sensitive wavelengths selected in the VIS–NIR using SPA, UVE, and CARS, which were 10, 93, and 51, respectively, accounting for 4.4%, 40%, and 22.3% of the full spectral range. From the figure, it can be seen that all three methods selected the pigment absorption regions in the 400–500 and 600–700 nm, indicating that color changes are related to SSC prediction. The 800–900 nm was also selected, suggesting that this region contains more spectral features relevant to SSC prediction. In Figure 3b,d,f represent the sensitive wavelengths selected in the SWIR using SPA, UVE, and CARS, with 20, 128, and 50 wavelengths, respectively, accounting for 4.4%, 40%, and 22.3% of the full spectral range. All three methods selected wavelengths in the 1000–1150, 1400, 1600, 1800, and 2100–2400 nm ranges or nearby. This indicates that these wavelengths contribute to SSC prediction.

Based on the sensitive wavelengths selected from both spectral ranges, PLSR models were established for the calibration set, and SSC predictions were made for the prediction set. The results are shown in Table 4. From Table 4, it can be observed that in the VIS–NIR, the model using the full‐wave spectrum provided the best prediction performance, with an R^2^_p_ of 0.827, an RPD of 2.403, and an RMSEP of 0.176. The prediction performance of the CARS method was the next best, with an R^2^_p_ of 0.815, an RPD of 2.325, and an RMSEP of 0.181. Compared to the full‐wave spectrum, the CARS method missed some key wavelengths, leading to a slight reduction in prediction performance. The UVE method showed moderate performance, with an R^2^p of 0.852, an RPD of 2.198, and an RMSEP of 0.192. Compared to the wavelengths selected by CARS, the UVE method selected a larger number of wavelengths but noticeably missed the green light region between 500 and 600 nm, which may have contributed to the decline in model performance. The SPA method selected the fewest wavelengths, resulting in weaker prediction performance, with an R^2^_p_ of 0.761 and an RMSEP of 0.201.

**TABLE 4:** Correction and prediction effect of PLSR model under different sensitive select methods on VIS–NIR and SWIR.

Wavelength	sensitive select	Latent variables	Calibration set		Prediction set		RPD
			*R* ^2^_c_	RMSEC	*R* ^2^_p_	RMSEP	
VIS–NIR (400‐970 nm)	Full wave	16	**0.868**	0.159	0.827	0.176	2.403
	SPA	7	0.842	0.174	0.761	0.201	2.045
	UVE	12	0.852	0.168	0.793	0.192	2.198
	CARS	15	0.859	0.164	0.815	0.181	2.325
SWIR (1000‐2400 nm)	Full wave	15	0.869	0.158	0.817	0.178	2.340
	SPA	9	0.827	0.182	0.801	0.186	2.242
	UVE	14	0.866	0.161	0.811	0.181	2.300
	CARS	20	0.878	0.153	0.818	0.177	2.344

**FIGURE 4:**
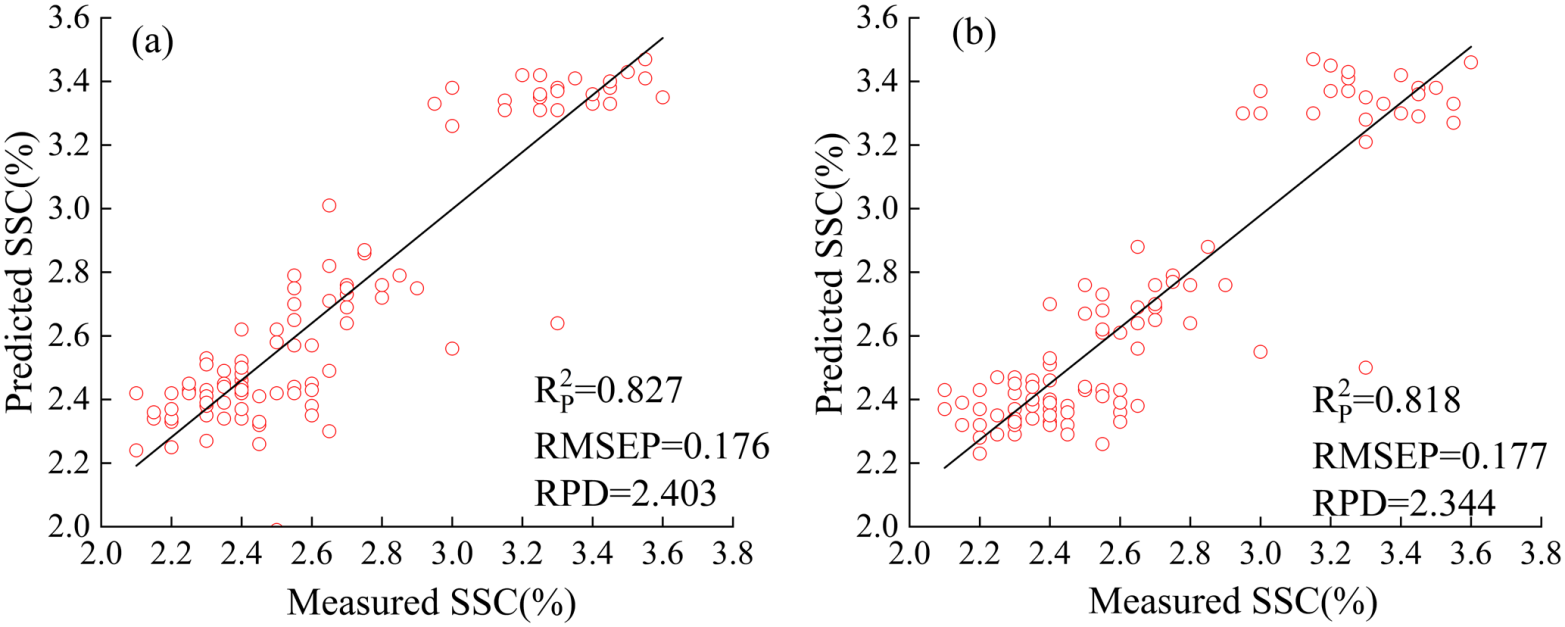
Prediction of set fitting effect (a) RAW‐SG‐Fullwave‐PLSR on VIS–NIR; (b) RAW‐MSC‐CARS‐PLSR on SWIR.

In the SWIR, the model based on the CARS‐selected wavelengths exhibited the best prediction performance, with an R^2^_p_ of 0.818, an RPD of 2.344, and an RMSEP of 0.177. This performance was better than that of the full‐wave spectrum, indicating that the 50 sensitive wavelengths selected by CARS preserved key information while significantly reducing data dimensionality, thereby enhancing the model's efficiency and practicality. The prediction performance of the UVE method was slightly lower than that of the CARS and full‐spectrum models, with an R^2^_p_ of 0.811, an RMSEP of 0.181, and an RPD of 2.3. Compared to the wavelengths selected by CARS, the 128 wavelengths selected by UVE included redundant wavelengths, such as those near 1700 nm, which led to a decrease in prediction performance. Figure 4 shows the fitting results of the prediction set for both the VIS–NIR and SWIR under SG‐Fullwave‐PLSR and MSC‐CARS‐PLSR.

## 4 Discussion

In this study, spectral data of fresh‐eating cucumbers from 7 to 28 days were collected using the VIS–NIR and SWIR imaging systems. In the VIS–NIR range, the cucumber spectra exhibited a fluctuating trend: an initial decrease, followed by an increase, and a final decline, with a peak at 700–800 nm. This consistency aligns with previous findings (Cen et al. 2014, 2016; Liu et al. 2005). In the SWIR range, we extended the cucumber spectral analysis to cover 1700–2400 nm, beyond the ranges used in previous studies (Ariana et al. 2006). The 2000–2400 nm range was found to contribute to SSC prediction, based on the selected sensitive wavelengths and corresponding model performance. This finding can serve as a reference for similar studies on other fruits and vegetables.

After spectral analysis of the VIS–NIR and SWIR, this study compares the two spectra in terms of preprocessing, sensitive wavelength selection methods, and SSC prediction model establishment. In the spectral data preprocessing process, the SG method significantly optimized the VIS–NIR, effectively improving the quality of the spectral data. The MSC method showed good optimization for the SWIR. This phenomenon is similar to the findings of Tantinantrakun et al. in their pineapple study (Tantinantrakun et al. 2023). Both cucumbers and pineapples have uneven surfaces, which can lead to light scattering in the SWIR. The MSC method can effectively eliminate spectral interference caused by scattering, improving the signal‐to‐noise ratio of the spectral data.

For the SSC prediction model, the PLSR model was used. By comparing different sensitive wavelength selection methods and model establishment, we discuss the following points. In terms of the full‐wave model performance, the full‐wave models of VIS–NIR and SWIR show differences, with the VIS–NIR outperforming the SWIR. This is consistent with research on grapes (Mejia‐Correal et al. 2023), strawberries (Amoriello et al. 2024), tomatoes (egei et al. 2022), and sweet peppers (Ignat et al. 2014). With the increase in cucumber growth, the color gradually changes from green to yellow, and soluble solids content gradually decreases. The VIS–NIR mainly involves O‐H and C‐H overtones and combination frequency absorptions. It also responds to pigment changes in the 400–700 nm range, indirectly contributing to SSC prediction. Regarding the impact of feature selection methods, the RPD values of the prediction models established under SPA, UVE, and CARS for the SWIR are similar, and the performance of different sensitive selection methods is stable. The MSC‐CARS‐PLSR is the best model, as CARS extracts sensitive wavelengths effective for predicting SSC (1000–1150, 1400 nm, 1600, 1800, 2000–2400 nm), greatly reducing the number of bands and computational cost. Lihongbo et al. also reported significant improvements in predicting kiwi soluble solids content using the CARS method (H. Li et al. 2024). In terms of practical applications for cucumber sorting factories, VIS–NIR is low‐cost and provides high prediction accuracy, making it more suitable as an online detection solution. Furthermore, in the VIS–NIR range, the PLSR model established using only the 10 wavelengths selected by SPA has an RPD of 2.045, and the prediction model also performs well. This provides a foundation for developing a low‐cost multispectral system for online cucumber SSC detection.

Hyperspectral imaging technology, as a powerful tool, can rapidly and non‐destructively evaluate internal quality parameters of cucumbers, such as SSC, which directly affect their market value and consumer satisfaction. The results of this study provide a theoretical foundation and technical support for improving the efficiency and accuracy of post‐harvest cucumber quality assessment. This is of significant practical importance for the post‐harvest commercialization industry, which faces an increasing demand for high‐quality cucumber products. However, there are some potential limitations in this study. First, the PLSR method was only used to model the VIS–NIR and SWIR, without fully exploring the potential of other advanced algorithms in spectral data analysis. Future research can explore multivariate modeling methods on the basis of determining the VIS–NIR or SWIR to improve the prediction accuracy and applicability of the models. Second, while this study focused on SSC, other important nutritional parameters such as moisture content, vitamin C, and tartronic acid were not considered. Future work should aim to integrate multiple quality attributes to provide a more comprehensive cucumber quality assessment. Finally, from a cost and real‐time perspective, future research could build on this study by using deep learning and large spectral datasets for multispectral reconstruction, followed by composition prediction or classification on the reconstructed multispectral data.

## 5 Conclusion

In this study, hyperspectral imaging technologies were used to collect reflectance spectra of cucumbers at different growth stages in the VIS–NIR and SWIR. Multivariate regression models for cucumber SSC were established in both wavelengths, enabling rapid detection of cucumber SSC. In the VIS–NIR, the SG‐fullwave‐PLSR model performed best in the prediction set (R^2^_p_ = 0.827, RMSEP = 0.176, RPD = 2.403). In the SWIR, the CARS algorithm was used to select spectral sensitive wavelengths, which effectively improved the model's accuracy and reduced complexity. The MSC‐CARS‐PLSR model was the optimal model (R^2^_p_ = 0.818, RMSEP = 0.177, RPD = 2.344). The results demonstrate that both VIS–NIR and SWIR hyperspectral imaging technologies can rapidly detect cucumber SSC. From the perspectives of prediction accuracy and cost, VIS–NIR is more suitable for online monitoring of cucumber SSC. Future research can focus on the application of VIS–NIR or SWIR for internal quality detection of cucumbers based on practical needs, considering factors such as detection accuracy, cost, and timeliness, and further optimizing modeling algorithms to enhance robustness and efficiency. The comparison of the VIS–NIR and SWIR also provides insights for the development of internal quality detection of other similar agricultural products based on spectral technologies.

## Author Contributions

**Fanghong Liu:** conceptualization (lead), methodology (lead), software (lead), writing – original draft (lead). **Ning Zhang:** conceptualization (supporting), investigation (lead), resources (lead). **Bo Huang:** conceptualization (equal), supervision (lead). **Xiujuan Chai:** funding acquisition (lead), supervision (equal), writing – review and editing (lead).

## Ethics Statement

The authors have nothing to report.

## Conflicts of Interest

The authors declare no conflicts of interest.

## Data Availability

The data that support the findings of this study are available from the corresponding author upon reasonable request.
